# Experimental evidence of a size-dependent sign change of the Seebeck coefficient of Bi nanowire arrays

**DOI:** 10.1038/s41598-023-35065-z

**Published:** 2023-05-22

**Authors:** Michael Florian Peter Wagner, Anna Sarina Paulus, Wilfried Sigle, Joachim Brötz, Christina Trautmann, Kay-Obbe Voss, Friedemann Völklein, Maria Eugenia Toimil-Molares

**Affiliations:** 1 Materials Research Department, GSI Helmholtzzentrum für Schwerionenforschung, 64291 Darmstadt, Germany; 2 Department of Materials and Earth Sciences, Technische Universität Darmstadt, 64287 Darmstadt, Germany; 3 Institute of Microtechnologies, RheinMain University of Applied Sciences, 65428 Rüsselsheim, Germany; 4 Max Planck Institute for Solid State Research, 70569 Stuttgart, Germany

**Keywords:** Nanoscale devices, Nanoscale materials, Nanoscience and technology, Materials science, Materials for devices, Materials for energy and catalysis, Nanoscale materials

## Abstract

The electrical transport in bismuth nanowires is strongly influenced by both sample geometry and crystallinity. Compared to bulk bismuth, the electrical transport in nanowires is dominated by size effects and influenced by surface states, which gain increasing relevance with increasing surface-to-volume ratios, i.e. with decreasing wire diameter. Bismuth nanowires with tailored diameter and crystallinity constitute, therefore, excellent model systems, allowing to study the interplay of the different transport phenomena. Here, we present temperature-dependent Seebeck coefficient and relative electrical resistance measurements of parallel bismuth nanowire arrays with diameters between 40 and 400 nm synthesized by pulsed electroplating in polymer templates. Both electrical resistance and Seebeck coefficient exhibit a non-monotonic temperature dependence, with the sign of the Seebeck coefficient changing from negative to positive with decreasing temperature. The observed behavior is size-dependent and is attributed to limitations of the mean free path of the charge carriers within the nanowires. The observed size-dependent Seebeck coefficient and in particular the size-dependent sign change opens a promising avenue for single-material thermocouples with p- and n-legs made from nanowires with different diameters.

## Introduction

The element bismuth (Bi), known since the eighteenth century, exhibits exciting and intriguing properties which are under investigation still today^1,2^. Its compounds are especially interesting within the field of thermoelectrics and have recently gained attention in the young research field of topological insulators, were it has been shown that Bi may belong to the class of higher order topological materials, proving that electrical transport processes of bismuth are still not completely unraveled^2–4^. This especially holds true for low-dimensional systems like nanowires, for which additional size effects and surface states can influence transport properties^5–8^.

Bulk Bi is a semimetal with a highly anisotropic Fermi surface. It possesses a low charge carrier concentration (~ 10^17^ cm^−3^) and a small effective mass^7,9^. The mean free path of the charge carriers is in the order of 100 nm at room temperature and as large as 400 µm at 4.2 K^10,11^. Therefore, finite size effects, e.g. the scattering of charge carriers on grain boundaries or at the surface in nanowires, described by models of Mayadas and Shatzkes as well as Fuchs-Sondheimer and Dingle, already occur at rather large nanowire diameters^12–15^. As a consequence of the small effective mass of the charge carriers also the Fermi wavelength of Bi is rather large with ~ 40 nm at room temperature^6^. When the geometric dimensions of the material are similar, this can lead to so called quantum-size effects, which affect the electronic density of states, and thereby the electronic transport properties of the material^5,6,16^. Due to the changes in electronic band structure, also a semimetal to semiconductor transition is exhibited in Bi nanowires as a function of nanowire diameter. Depending on the crystalline orientation the transition occurs at diameters of ~ 40 to 55 nm diameter at 77 K^8,17^.

The predicted quantum-size effects drew the interest of the thermoelectrics community as they seemed to offer a way to significantly increase the Seebeck coefficient (*S*), i.e. the voltage generated by a material if a temperature difference is present, in very thin nanowires compared to bulk material^7,16,18,19^. The initially predicted gains could however not be realized and newer calculations by Cornett et al. taking into account the contribution of multiple subbands to the transport showed that gains in the power factor would only be achieved for even thinner nanowires with wire diameters smaller than 17 nm^20^. Calculations by Kim et al. applying the Landauer formalism also showed that while the Seebeck coefficient per mode can be improved by lower dimensionality a large packing density of nanowires with small diameters is required to realize this advantage^21^. However, even though the Seebeck coefficient may increase, at those small wire sizes the electrical conductivity decreases due to a stronger sensitivity to carrier scattering mechanisms and geometry, possibly preventing to achieve total gains in the power factor^22^. In addition, even the Seebeck coefficient gains theoretically predicted for diameters smaller than 10 nm might not be realized due to significant contributions of surface states to the electrical transport within Bi. Since the surface-to-volume ratio for nanowires can be extremely high, effects due to surface transport are also expected to significantly contribute to the overall transport properties of nanowires^23^. Metal like surface states for low indexed planes in Bi are known, rendering the surface considerably better metallic behavior than the bulk^9^. To what extend also topological surface states in bismuth could contribute is still under investigation^2–4^. In general, both kinds of surface states can behave metal-like and thereby prevent an increase in Seebeck coefficient^3^. To provide further insights on the special combination of unique transport processes with opposite contributions to the Seebeck coefficient and electrical resistance, Bi nanowires with tailored diameter from bulk-like down to few nanometers and controlled crystallinity present a unique model system to study the interaction and interplay of these different size-dependent processes.

Over the years several fabrication methods for Bi nanowires have been developed including hydro- and solvothermal synthesis, stress-induced growth, vapor liquid solid methods, the Taylor process and the so-called template method, which is applied here^24–32^. For the template method first a mold is prepared which is subsequently filled with the desired material. This enables an excellent control of the samples geometrical parameters, such as nanowire diameter, density number and alignment of nanowires, determined by the properties of the hosting template, while the filling process determines composition, crystallinity, crystal orientation and length^31–33^.

Template materials most commonly used to fabricate nanowires are either porous anodic alumina oxide (AAO) or ion track-etched polymer membranes^33,34^. The alumina templates usually allow the fabrication of longer nanowires (> 120 µm) that are ordered into hexagonal patterns. On the other hand, the polymer membranes used in this work are more chemically resistant and therefore allow the use of a larger variety of electrolytes ranging from strong acidic to alkaline solutions. Also the removal of the polymer can be achieved more easily without damaging the nanowires. It is known that the alkaline solutions required to dissolve AAO oxidize the nanowires surface, whereas polycarbonate (PC) membranes can be removed using organic solvents that in most cases do not affect the surface of the wires. Additionally, the thermal conductivity of polymer membranes is lower than that of AAO, which makes thermoelectric transport measurements and later applications more feasible^35,36^. Pore filling methods developed for AAO or polymer templates include chemical vapor deposition, pressure injection or electroplating. Potentiostatic electrodeposition of Bi has been used in the past by our group and others for the synthesis of nanowire arrays^11,12,37–39^. Recently, we reported that pulse plating leads to a more homogeneous growth of Bi nanowire arrays over larger deposition areas, when the potential is switched periodically between a reduction potential (on-time) and a potential were no reaction occurs (off-time)^31,32,39^. In this case, for parallel nanowire arrays, homogeneous growth was obtained both at 22 °C and 40 °C using an aqueous-based electrolyte without and with organic additives, respectively^31,32^. In the case of 3D interconnected nanowire networks, we recently reported that the addition of a small percentage of organic surfactant (~ 1‰) to the electrolyte was necessary to achieve a homogeneous growth and filling in the interconnected nanochannels over the entire deposition area. It is known that the addition of organic additives can significantly influence the crystallinity of the deposits^40^. In this work we systematically investigate whether the addition of surfactant to the electrolyte influences the crystallinity, morphology, as well as Seebeck coefficient and electrical resistance of Bi nanowire arrays. Parallel arrays of Bi nanowires with diameters between 30 and 400 nm are fabricated in ion track-etched polycarbonate templates by pulsed electrodeposition at 22 °C without surfactant and at 40 °C with surfactant. The morphology and crystallinity of the wires after plating are also discussed based on scanning electron microscopy (SEM), X-ray diffraction (XRD), as well as transmission electron microscopy (TEM) data. Subsequently their Seebeck coefficient and electrical resistance are measured as a function of wire diameter and temperature. These results show experimental evidence of the size-dependent sign-change of the Seebeck coefficient, as previously predicted by Murata et. al.^47^.

## Results and discussion

The electrodeposition of nanowires in the nanochannels of etched polycarbonate ion-track membranes depends of many parameters, including voltage (*U*), temperature (*T*), and electrolyte composition, as well as the geometrical characteristics of the template, such as nanochannel diameter, length, density, and orientation^31–33,42^. Thus, to subsequently perform systematic transport measurements e.g. as a function of nanowire diameter, a thorough systematic structural characterization of the electrodeposited nanostructures are required.

The samples were prepared by pulsed electroplating of Bi from an aqueous solution into the pores of an ion track-etched polymer membrane using two different sets of parameters: (i) at 22 °C without addition of surfactant dowfax 2a1 and (ii) at 40 °C adding one per mile Dowfax 2a1 surfactant to the electrolyte. The electrodeposition curves and the subsequent characterization of the nanowires by X-ray diffraction (XRD) revealed that differences in the crystalline structure of the nanowires were mainly caused by the difference in plating temperature, while the addition of surfactant in such small quantities did not affect their texture. Thus, for clarity, we will refer to the two processes by specifying the deposition temperature. For more details, please see the supplementary information Fig. S1 and S2.

**Figure 1:**
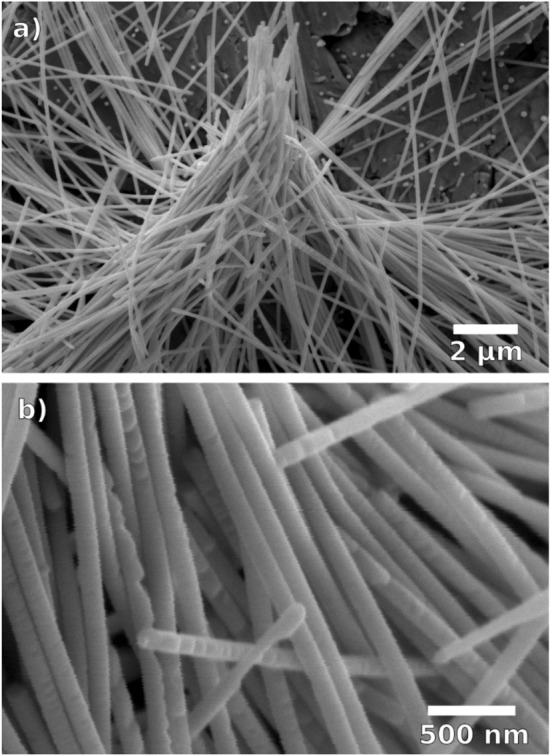
Representative SEM images of Bi nanowire arrays (length ~ 30 µm, wire diameter ~ 100 nm) grown with surfactant at 40 °C.

Figure 1 shows representative scanning electron microscopy (SEM) images of ~ 100 nm diameter Bi nanowire arrays electrodeposited at 40 °C. After removal of the polymer matrix, the wires cannot support their weight and collapse onto each other forming tipi-like structures, displaying well the flexibility and length of the grown nanowires (Fig. 1a). In general, the cylindrical nanowires display both smooth parts as well as sections with pronounced indents. Such indents had been previously observed Bi_2_Te_3_ nanowires electrodeposited at high overpotentials^41^. Smoother nanowires are expected at smaller overpotentials.

**Figure 2:**
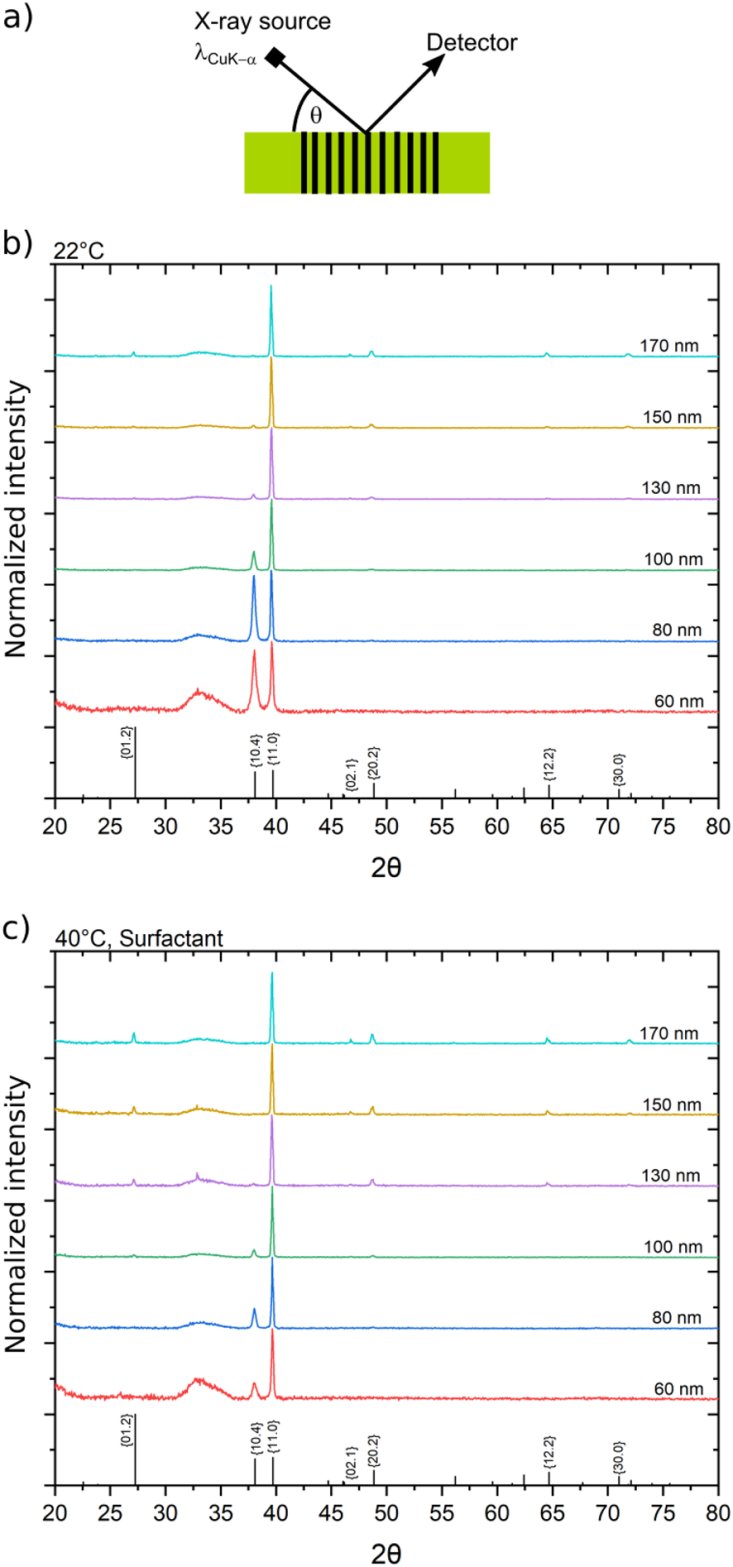
(**a**) Schematic of the XRD measurements in reflection. Diffractograms of Bi nanowire arrays with different average nanowire diameters grown at 22 °C (**b**) and 40 °C (**c**). The corresponding average wire diameters are given on the right side. At the bottom, powder reference data based on simulations by VESTA, using crystal structure data by P. Cucka and C.S. Barrett are shown^62,63^.

Figure 2 displays the XRD patterns recorded in reflection of Bi nanowire arrays with various average wire diameters embedded in polymer. Here, the hexagonal notation is applied, using a “.” instead of “*i*” which can be calculated by *i* = − (*h* + *k*), and all diffractograms are normalized to the {11.0} reflexion. Prior to the XRD measurements the Au contact on the template was removed using Lugols iodine solution. All of the samples possess a strong {11.0} texture, i.e. a preferential orientation of the crystals with {11.0} planes perpendicular to the wire axis. Large diameter nanowires exhibit a larger number of reflexions. With decreasing wire diameter the intensity of the {10.4} reflexion increases relative to the {11.0} reflexion while other reflexions vanish (e.g. {10.2}{12.2},{20.2}). For the smallest nanowires with 60 nm wire diameter only two reflections, {10.4} and {11.0}, are observed. This trend is observed for the samples grown at 22 (Fig. 2b) as well as 40 °C (Fig. 2c), the {10.4} reflection being less prominent for samples grown at 40 °C, which can be explained by the faster growth of the nanowires at this temperature (Fig. 6). For comparison, Bi nanowires grown by Cassinelli et. al., using the same electrolyte composition without surfactant and applying the pulsed potential of *U*_ON_ = −220 mV, *t*_ON_ = 20 ms, *U*_OFF_ = −150 mV, *t*_OFF_ = 100 ms, vs. SCE at room temperature also exhibited a {11.0} texture, but with significantly higher intensity for the {01.2} reflexions.^32^ The broad band and reflexion visible at angles between ~ 30° and 37° is due to the Si substrate on the sample holder.

The crystallinity of 80 nm diameter wires was additionally investigated by transmission electron microscopy (TEM) and selected area electron diffraction (SAED). Representative images are shown in the Fig. S3 within the supplementary information. For both nanowires grown at 22 and 40 °C, several µm long single-crystalline sections were observed. These results confirm that the applied pulsed plating conditions, carefully chosen to enhance the diffusion of electrolyte towards the working electrode during the OFF pulses led to almost single-crystalline growth and avoided grain refinement. It should however be noted that nanowires with larger wire diameter may consist of a larger number of grains with different orientations, as indicated by the additional reflexions in the diffractograms^42–44,59^. In general, the wires possess a texture with small angle rotation grain boundaries in between grains. Growth directions along the wire axis determined from SAED patterns are consistent with the directions observed in XRD. In summary, whereas the addition of surfactant had no significant effect on the nanowires, the effect of pore diameter and temperature on the crystallinity of the wires has been evidenced and will be taking into account for the interpretation of the Seebeck measurements.

**Figure 3:**
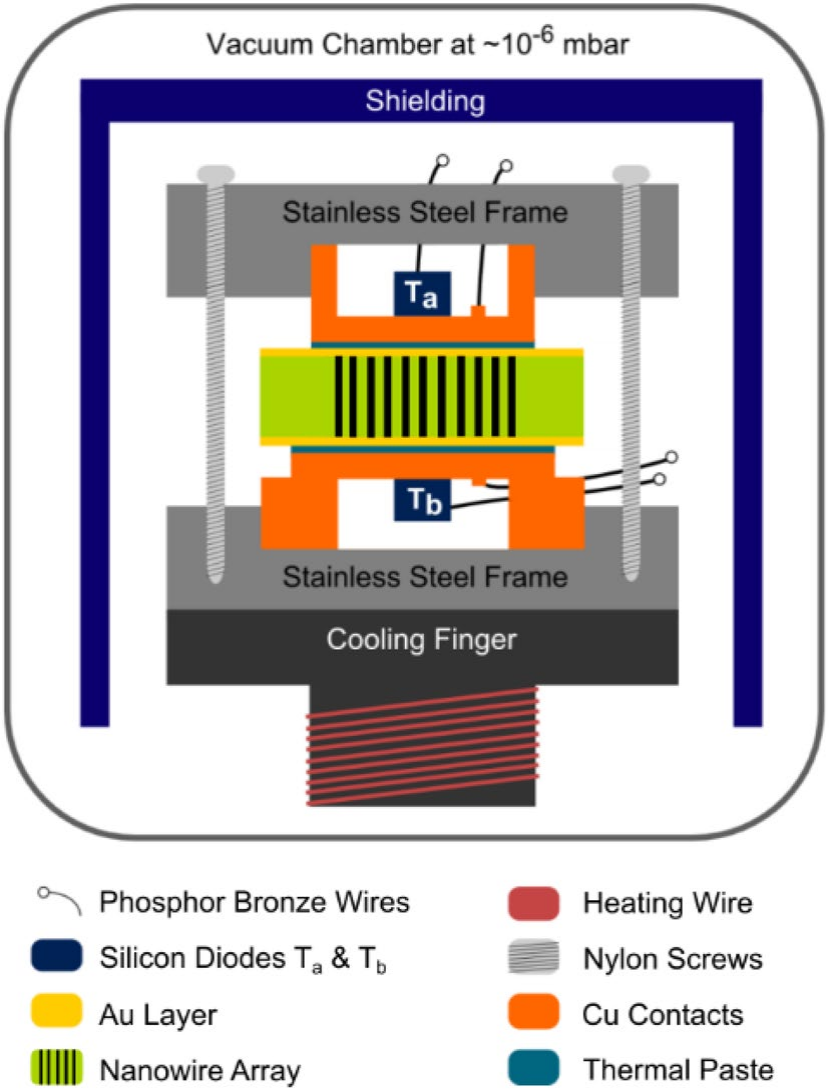
Schematic of the setup used for the measurement of the Seebeck coefficient and relative electrical resistance^19,45,46^.

In order to measure the electrical resistance and Seebeck coefficient, the nanowires embedded in the PC matrix were placed in a custom build setup, shown in Fig. 3^19,45,46^. Each sample (black wires embedded in the green polymer) are provided with two Au layers (yellow layers), one on each side, and electrically contacted by two copper blocks (orange), allowing the determination of the electrical resistance by *I–V* measurements. For the measurement of the Seebeck coefficient a temperature difference Δ*T,* (*T*_a_ – *T*_b_), is generated between the two copper blocks, i.e. along the nanowires and the generated thermal voltage *V*_*Th*_ is measured. The Seebeck coefficient of the nanowire arrays is calculated using the measured thermal voltage *V*_*Th*_ and temperature difference Δ*T* between top and bottom. More details are provided in the experimental section.

**Figure 4:**
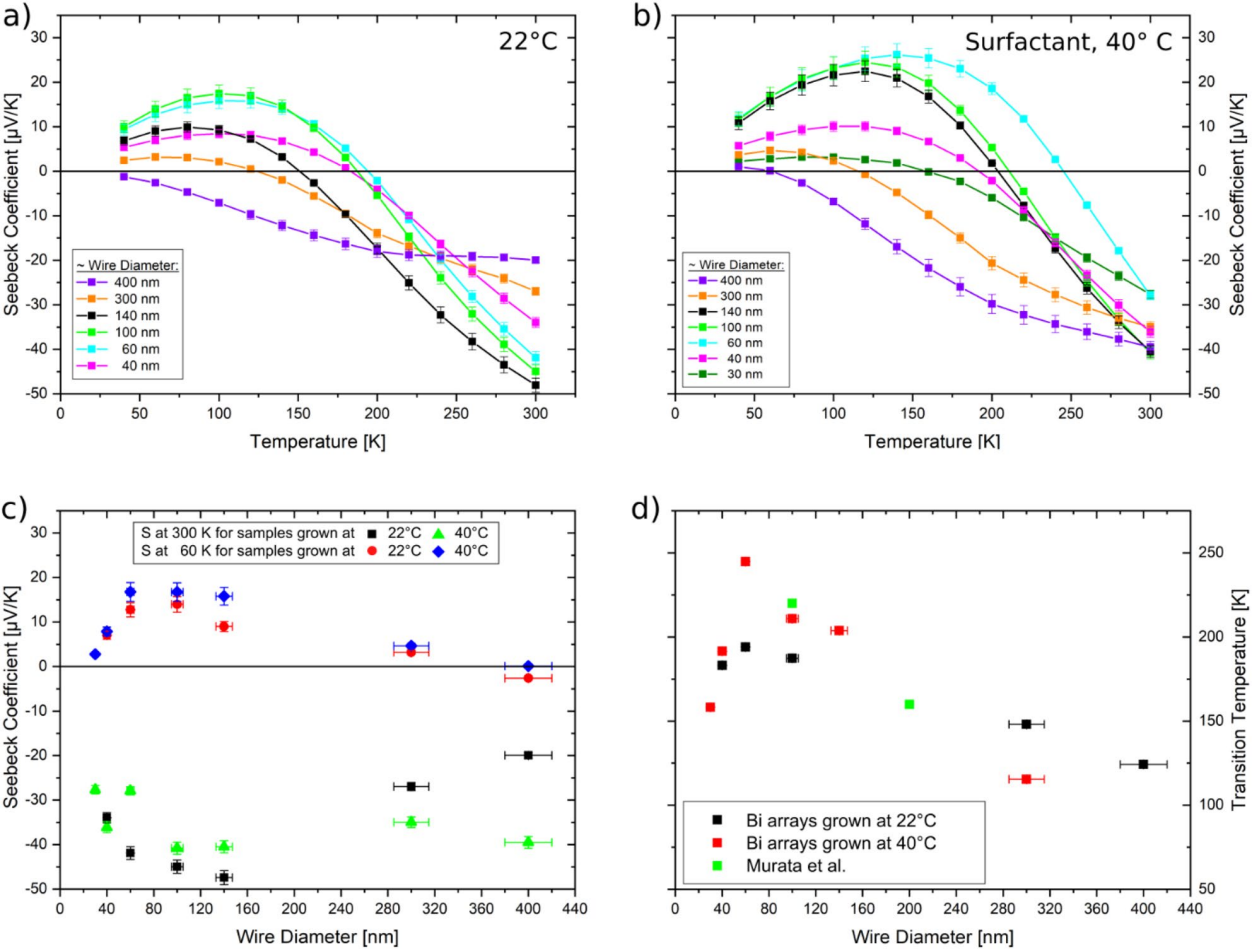
Seebeck coefficients of Bi nanowire arrays prepared (**a**) at 22°C and (**b**) at 40 °C as a function of temperature. Lines between data points serve as a guide-to-the-eye. (**c**) Seebeck coefficient as a function of nanowire diameter at 300 K (black squares and green triangles) and at 60 K (red circles and blue diamonds) ambient temperature. (**d**) Transition temperature (negative to positive S) as a function of wire diameter. The graph also contains theoretical values taken from the work of Murata et al. (green)^41,45^.

Figure 4 shows the Seebeck coefficient as a function of temperature of Bi nanowire arrays with diameters between 40 and 400 nm electrodeposited (a) at 22 °C and (b) at 40 °C. At 300 K the Seebeck coefficient of all samples is negative as expected for n-type Bi and depends on the nanowire diameter. Figure 4a, b evidence that for the majority of samples, the absolute value of *S* decreases with decreasing temperature exhibiting a sign change from positive to negative at a temperature *T*_np_. The 400 nm diameter wires grown at 22 °C are the only sample that did not exhibit a sign change within the measured temperature range. Correspondingly, as shown in Fig. 4c, at 60 K *S* is positive for all samples except for the 400 nm diameter nanowires. A sign change of the Seebeck coefficient in Bi nanowires has been also observed by other groups.^5,41,49–51^ In most cases after the sign change *S* first increases before it moves towards zero again with decreasing temperature. Figure 4c shows the *S* measured at 300 K as a function of nanowire diameter for the samples grown at 22 °C (black squares) and at 40 °C (green squares). The S values measured at 60 K are also plotted (blue and red symbols, respectively), exhibiting a non-monotonic dependence on the nanowire diameter. For all nanowire diameters, the values at 300 K are lower than the reported maximum bulk values, which at 300 K is in the order of −120 µV/K for the trigonal direction and around −60 µV/K for the binary and bisectrix direction^47^. Considering that the nanowires show a strong texture along the (11.0) and/or (10.4) direction instead of one of the principal directions, the measured S values are in reasonable accordance with values reported for other Bi nanowires in literature. These values typically range between −20 and −80 µV/K at 300 K depending on orientation and wire diameter.^7,19,41,48,49^ The *S* value first increases when decreasing the wire diameter from 400 nm down to 140 nm, and then decreases for nanowire diameters from 140 nm down to 30 nm. For nanowires with diameters larger than ~ 140 nm (i.e. larger than the critical length scales) quantum-size-effects are not expected and thus neither band edge- nor Fermi-energy changes^7,20,22^. The decrease in S for larger diameters is more pronounced for the wires grown at 22 °C, for which also stronger changes in texture were determined from the XRD measurements^13–15,41^.

Figure 4c, d evidence that the absolute Seebeck coefficient at 300 K decreases and T_np_ shifts to higher *T* with decreasing wire diameter. This behavior was theoretically predicted by Murata et al. for Bi wires aligned along the bisectrix orientation^41^. Murata et al. investigated how the limitation of the mean free path of charge carriers in nanowires due to increasing scattering at the nanowire surface affects the mobility of the charge carriers and thereby the electrical resistivity and Seebeck coefficient^41^. Contrary to quantum-size-effects, finite-size-effects due to surface scattering are already expected at rather large wire diameters in Bi as the mean free path for electrons and holes for pure bismuth is comparable and in the order of 100 nm at room temperature^10,11^.

According to Murata et al. the Seebeck coefficient for Bi nanowires can be calculated by

$$S=\frac{{S}_{e}b+{S}_{h}}{b+1},$$

with *S* being the total Seebeck, *S*_*e*_ and *S*_*h*_ being the Seebeck coefficient of electrons and holes respectively and *b* = µ_e_/µ_h_ being the ratio of the mobilitys of electrons and holes. The mobilitys depend on temperature, wire diameter and crystal orientation. While for the trigonal direction e.g. there is little effect of the wire diameter on the Seebeck coefficient and no sign change is observed with decreasing temperature, for the bisectrix orientation a decreasing Seebeck coefficient with decreasing wire diameter as well as a sign change is expected. Assuming identical charge carrier concentrations for holes and electrons, the sign change of the Seebeck coefficient happens when the mobility of holes becomes larger than the mobility of the electrons, which can occur because the mobility of electrons and holes is affected differently by surface scattering due to their different effective masses *m*_*eff*_^41^. A similar explanation was given by Nikolaeva et al. for the occurrence of a sign change of *S* in Bi, which suggested that the electron mobility becomes smaller than the hole mobility in the low temperature range, because the hole mobility was not significantly affected by boundary scattering^51^.

Figure 4d presents the transition temperature *T*_*np*_ as a function of nanowire diameter. *T*_np_ was determined from the intercept of the line connecting the two closest data points above and below the 0 µV/K line with said line for each sample in Fig. 4a, b. For wire diameters from 400 down to 60 nm the transition temperature increases linearly with decreasing wire diameter, which is consistent with theoretical predictions by Murata et al. (green squares) for transport along the bisectrix direction^41^. For smaller wire diameters (40 and 30 nm) however the transition temperature increases again with decreasing wire diameter, which was not described by Murata et al., as their work did not predict the behaviour for nanowires smaller than 100 nm. For wire diameters in the order of ~ 50 nm several additional effects including a semimetal-to-semiconductor transition and surface states becoming the majority charge carriers can play a role^9,48,49,52^.

In the future, additional measurements will be pursued to determine charge carrier densities, mobilitys and surface states densities on single nanowires. For example, challenging angle resolved X-ray spectroscopy (ARPES) on single nanowires can be applied to directly determine the band structure and contribution of surface states^53^. Using ARPES Agergaard et al. estimated the carrier density of the surface states on Bi(110) surfaces to be in the range of 5 × 10^12^ cm^−2^^54^. Cassinelli et al. reported a decrease in absolute Seebeck coefficient with decreasing wire diameter from ~ 140 to 60 nm and a following increase from 60 to 30 nm, for {11.0} and {01.2} textured nanowires and attributed this behaviour to the complementary contribution of both bulk and metallic surface states to the thermopower, assuming an increasing influence of the surface states as the diameter decreases and the surface to volume ratio increases^3,23,32^.

For bulk bismuth the electrical resistance is known to decrease monotonically with decreasing temperature as the increase in charge carrier mobility, two orders of magnitude larger than the changes in charge carrier concentration, dominates the transport response^7,10,47,55^ As mentioned above, due to the large mean free paths of charge carriers in bismuth however, the resistance behaviour for nanowires is significantly altered due to additional surface scattering of charge carriers, which can affect nanowires even larger than 5 µm diameter^7,41^.

**Figure 5:**
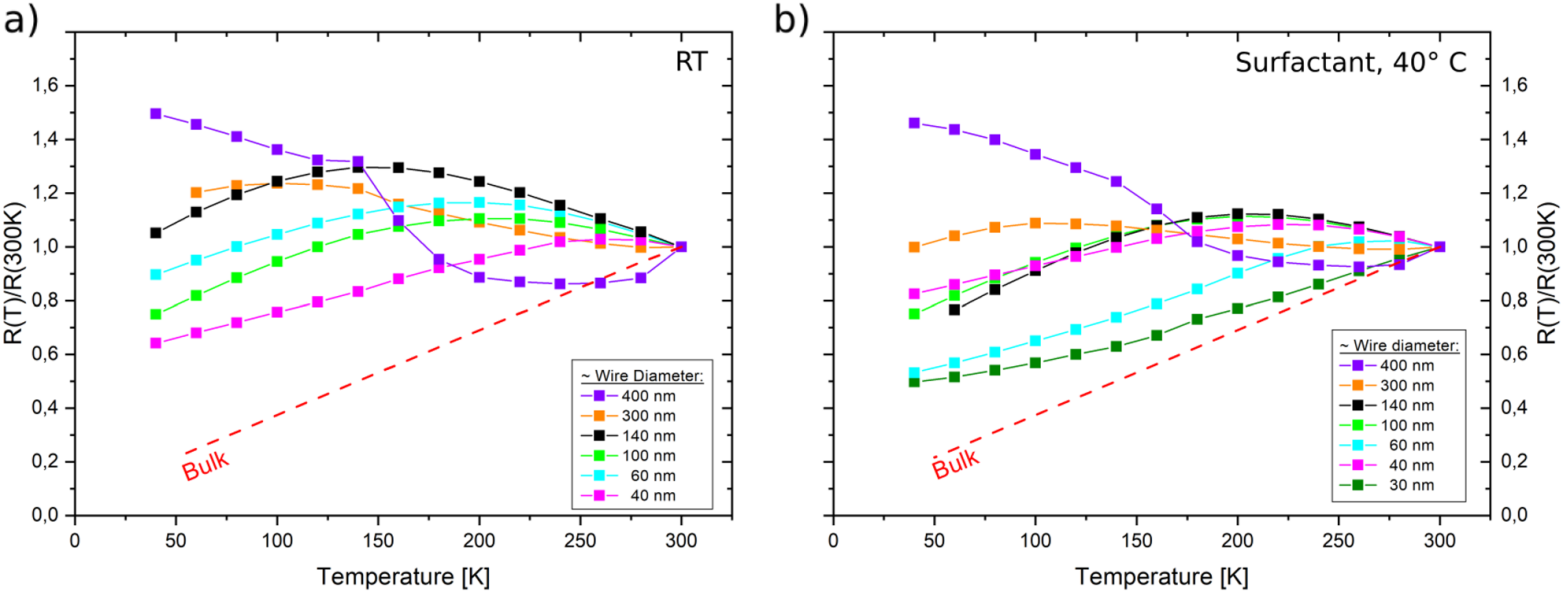
Relative resistance of Bi nanowire arrays with diameters between 30 and 400 nm prepared **a**) without surfactant at RT (22°C) and **b**) with surfactant at 40 °C. The values for bulk resistance were taken from^47^. Lines between data points serve as a guideline to the eye.

Figure 5 shows the electrical resistance of the Bi nanowire arrays normalized to the resistance measured at 300 K (*R*_*T*_*/R*_*300*_). As the exact number of contacted nanowires is unknown and can vary from sample to sample, only relative resistance values are reported here. With the exception of nanowires with 30 nm diameter where the relative resistance shows a monotonous decrease, nanowires with larger diameters show a non-monotonous dependence of the relative electrical resistance with temperature. For nanowires smaller than 400 nm diameter a maximum in resistance is observed that tends to shifts to higher temperatures with decreasing nanowire diameter, whereas for 400 nm diameter nanowires no maximum is observed within the measured temperature range.

For Bi nanowires fabricated by various methods a non-monotonic behaviour of the resistance has been reported, which is ascribed to the complex interplay between charge carrier density and charge carrier mobility, both dependent on temperature, crystallinity, crystal orientation and nanowire diameter^7,19,41,55,56^. The influence of classical size effects like the scattering of charge carriers at the surface and grain boundaries on the mobility has previously been described by models of Fuchs, Mayadas and Dingle^13–15,57,58^. Additionally, it has been previously reported, that in the case of a non-monotonic *R* versus *T* behavior, the resistance maximum shifts to lower temperatures for nanowires consisting of smaller grains^59^. From previous studies on Bi nanowire arrays, it is known, that the grain size decreases with increasing nanowire diameter^59^. Thus, for Bi nanowires consisting of sufficiently small crystals the resistance becomes a monotonic function of temperature, as reported by Liu et al.^56^.

In this work, the non-monotonic *R*-vs.-*T* behaviour is attributed to opposite contributions of an increasing mobility and a decreasing charge carrier density with decreasing temperature. The gain in mobility is limited by additional surface scattering and the nanowire’s resistance does not exhibit the monotonous decrease as the bulk material. With decreasing nanowire diameter, the effective grain size increases (as known form TEM and XRD results that evidenced longer grains and higher texture coefficients, respectively), shifting the *T* at which *R*_*T*_*/R*_*300*_ is maximal to higher values. For the smallest wire diameters, which consist of only few elongated single-crystals, a monotonous decrease of the resistance with decreasing temperature is observed. Alternatively, it is possible that surface states also significantly contribute to transport, which could also explain the metallic behaviour of the very small diameter nanowires. The role of surface states will be tackled, as explained above, in future measurements, applying additional characterization methods.

## Conclusions

Bi nanowire arrays have been synthesized by pulsed electroplating employing a BiCl_3_-based aqueous electrolyte. In particular, the growth and the resulting crystallographic properties of Bi nanowire arrays electrodeposited at 22 °C and 40 °C, with and without surfactant have been systematically investigated. The additional surfactant does not have a significant influence on the growth and resulting structure, while the temperature does. In both cases, the Bi nanowires exhibited cylindrical geometry with a smooth contour, sometimes interrupted by sections exhibiting indentations, associated to the crystal growth. The XRD patterns of the samples showed a preferred {11.0} texture for large diameter nanowires, with an increasing additional {10.4} texture with decreasing wire diameter. TEM measurements revealed that individual nanowires of 80 nm diameter consist of single crystalline segments of several µm length (i.e. bamboo-like structure), and confirmed the preferred crystallographic orientations determined by XRD.

The Seebeck coefficient and the relative electrical resistance of nanowire arrays synthesized under both sets of electrodeposition conditions, with wire diameters varying between 30 and 400 nm, were measured systematically down to 40 K. The Seebeck coefficient of both types of samples exhibited a similar behavior, a non-monotonic dependence with temperature, displaying a sign change from a negative to a positive Seebeck coefficient as the temperature decreased. The observations were attributed to mean free path limitations of the mobility of the charge carriers within the wires, previously theoretical predicted by Murata et al. and potential additional contributions of surface states in case of smaller nanowires^19,23,41^. The electrical resistance of Bi nanowires displays the well-known non-monotonic behavior as a function of temperature, that is ascribed to the complex interplay between charge carrier density and charge carrier mobility in nanomaterials, previously described by Fuchs, Mayadas and Dingle^13–15,57,58^. The thinnest nanowires exhibited a monotonic decrease with decreasing temperature which may indicating the contribution of surface states with metallic behaviour. The observed size-dependent Seebeck coefficient, and in particular the size-dependent sign change evidences once more the complexity of transport phenomena in Bi nanostructures, and opens a promising avenue for single-material thermocouples with p- and n-legs made from nanowires with different diameters.

## Methods

### Template fabrication

Templates were prepared by first irradiating 30 µm thick polycarbonate foils (Makrofol N, diameter 30 mm, thickness 30 µm) with ~ 2.2 GeV Au ions at the UNIversal Linear ACcelerator (UNILAC) at the GSI Helmholtz Center for Heavy Ion research in Darmstadt, Germany. Each foil was irradiated with ~ 1.5 × 10^8^ ions/cm^2^. Due to the high energy deposited by each individual ion along its trajectory a so-called latent track is generated^60^. The irradiated foils were afterwards exposed to UV-light (T-30M Vilber Lourmat lamp, 30 W, 312 nm) for an hour on both sides. This is known to narrow the distribution of pore diameters after the following etching process^61^. In the final step the ion tracks were selectively etched by immersing the irradiated foils in 6 M NaOH solution (NaOH purity ≥ 98%) at 50 °C^33^. Using these conditions cylindrical pores are obtained, whose diameter was controlled via the etching time. Under these conditions the etching rate of the pores was 10 ± 1 nm/min. After etching, the templates were rinsed in several DI-water baths, then stored in DI-water for a day in order to completely remove leftover NaOH from within the pores, and finally dried on air.

### Nanowire growth

**Figure 6:**
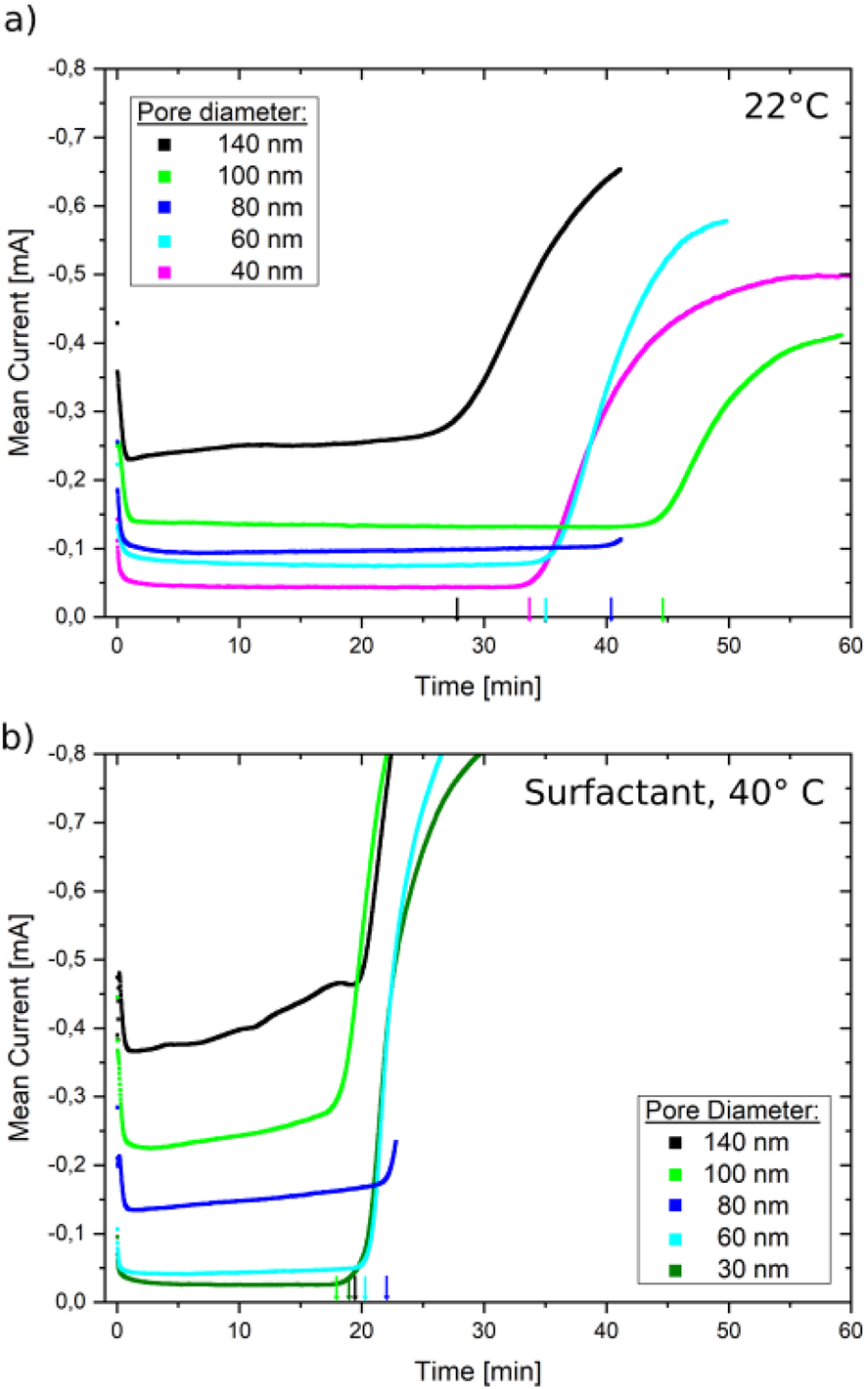
Mean current vs. plating time for platings (**a**) without surfactant at 22 °C and (**b**) with surfactant at 40 °C, respectively. The colored arrows on the horizontal time axes mark the plating time when cap growth started for the corresponding sample.

First, a ~ 100 nm thick gold layer was sputtered onto one side of the template to provide an electrical contact to the sample. This layer was subsequently reinforced by electroplating an additional one µm thick Au layer. The electroplating of Au was done at 22 °C using a two electrode setup, with the sputtered Au layer acting as cathode (working electrode) and a Au spiral as anode (counter electrode). A potential *U* = *−*0.7 V was applied using a GAMRY Reference 600 potentiostat. This additional electroplated Au layer closed off all pores that were eventually still open after sputtering, so that no electrolyte could leak through to the backside of the sample during nanowire plating, and guaranteed a good mechanical stability of the samples even when the polymer matrix is removed. For the electroplating of Bi, an aqueous electrolyte consisting of 0.1 mol/L Bi(III)-chloride (for analysis quality), 0.3 mol/L tartaric acid (99.5% purity), 0.2 mol/L NaCl (for analysis quality), 1.95 mol/L HCl (99.5% purity), and 1.09 mol/L glycerol (99.5% purity) was used^31,32^. For a second series of experiments 1 ml/L Dowfax 2A1 surfactant was added to the electrolyte^39,45^. A three electrode setup consisting of a Pt_80_Ir_20_ spiral as counter- and a standard calomel electrode with saturated KCl (SCE) (Sensortechnik Meinsberg) as reference electrode was used. We applied the same pulsed ON/OFF potential (− 200 mV for 20 ms/− 170 mV for 100 ms) as previously used to homogeneously grow Bi nanowire networks^39,45^. In the cases were the solution contained surfactant, the plating was performed at 40 °C, otherwise the plating was done at 22 °C^19,31,32^. During the electrodeposition the current between working and counter electrode was recorded, exemplarily shown in Fig. 6. When a significant increase in current was observed the electroplating was stopped, as this indicated the formation of so called caps on top of the sample, which appear when the growing nanowires reach the top of the pores^33^.

The nanowire electrodeposition with surfactant at 40 °C for different pore diameters takes ~ 20 min till cap growth which is a bit more than half the time of the average plating without surfactant at 22 °C (Fig. 6a). The faster growth rate (~ 1.5 µm/ min vs ~ 0.8 µm/min) is solely a consequence of the higher plating temperature, which increases the reaction rate and thereby the current during electroplating^40^.

The filling ratio (i.e. how many of the pores are filled) is estimated by comparing the charge employed for nanowire growth till cap growth versus the charge required to fill the whole pore volume in a template with Bi. The charge deposited during electroplating is given by integrating the I–t curve from 0 until the time of cap growth. The beginning of cap growth is indicated with vertical lines on the time axis in Fig. 6. For the calculation it is assumed that 100% of the charge is used for the electroplating of Bi and no side reactions occur. The theoretical charge required for filling all pores is calculated by using Faraday’s laws of electrolysis using pore densities and pore diameters measured by SEM to estimate the total pore volume that needs to be filled with material^40^. Average filling rates at 22 °C were (90 ± 5)% versus (70 ± 5)% at 40 °C.

### Sample characterization

To investigate the morphological and crystalline properties of the nanowires, SEM, XRD, as well as TEM was carried out. XRD measurements were done within a Bragg- Brentano geometry while the nanowires were still embedded in the PC template. To index the reflexes of Bi, crystal structure data of Bi measured by P. Cucka and C. S. Barrett was used to simulate the powder diffraction pattern using VESTA^62,63^. SEM and TEM measurements were done on wires after the polymer matrix was dissolved in several baths of DCM. In case of the TEM measurements the nanowires were separated from the metal electrode by ultra-sonicating the solution and subsequently drop-casting the DCM/nanowire mixture onto Cu-lacey TEM grids.

### Seebeck and electrical resistance measurements

For electrical transport measurements a custom build setup shown in Fig. 3, was used^19,45,46^. Prior to the measurements an additional ~ 400 nm thick layer of Au was sputtered onto the cap side of the sample to allow good electrical contact to the nanowires. The sample was then placed between two copper blocks within a vacuum chamber which was operated at 10^–6^ mbar during measurements to prevent heat loss due to convection. The copper blocks served as electrical contacts to the sample, as well as heat sinks. During measurements the bottom copper block was constantly cooled and periodically heated allowing measurements at ambient temperatures between 300 and 40 K, which were changed in steps of 20 K. Due to the heating and cooling procedure, the temperature of the bottom heat sink oscillated by ~ 2 K around the chosen ambient temperature. As the heat sink on top of the sample is thermally only directly connected to the sample, its temperature response is delayed when compared to the bottom heat sink. The thereby generated temperature difference Δ*T* then induces a thermoelectric voltage *V*_*Th*_ within the sample. The thermoelectric voltage of the sample as well as the temperature of both copper blocks, given by diodes attached to them, was measured every 10 s using a nanovoltmeter and a temperature controller, respectively. The Seebeck coefficient was then calculated by taking the slope of a *V*_*Th*_*–*Δ*T* graph. To determine the electrical resistance of the sample *I–V* curves were measured after the thermal voltage and temperature were measured. As the 4-point probes used were connected to the copper blocks these measurements also included the contact resistance between the copper blocks and the sample. In total two measurement runs were carried out. The first one was done as described above and was used to determine the relative electrical resistance of the sample. For the second run an additional thermal paste (PK-1 Thermal Compound by Prolimatech) was applied between the sample and the copper blocks. This was done to reduce the thermal resistance between copper blocks and sample in order to get a more precise reading of the temperature difference and thereby a more exact value for the Seebeck coefficient^19,45^.

As the exact number of nanowires is unknown and significant contact resistance in comparison to the nanowire arrays were observed, only relative electrical resistances were investigated. The error of the relative electrical resistance is assumed small (< 1%) and defined by the instruments used. The error of the Seebeck coefficient is mainly given due to uncertainties of the actual and measured temperature difference along the sample. Measurements of the Seebeck coefficient without thermal paste were up to 55% smaller than with paste depending on the ambient temperature. Assuming that the thermal paste paste causes the temperatures measured to be similar to the actual ones along the sample the error of the temperature difference is based on error of the diodes which is in the order of 3–13% for the temperature difference and depends on the ambient temperature, with the error being larger at lower temperatures.

## Supplementary Information

Supplementary Information.

## Data Availability

The data that support the findings in this study are available from the corresponding author upon reasonable request.
