# Supplementary material for: Experimental evidence of a size-dependent sign change of the Seebeck coefficient of Bi nanowire arrays
Source: Sci Rep. 2023 May 22;13:8290. doi: 10.1038/s41598-023-35065-z (PMC10203367; doi:10.1038/s41598-023-35065-z)
Supplement: Supplementary file 1 — Supplementary Information. [file 41598_2023_35065_MOESM1_ESM.docx]

### Supplementary information

### **Effects of Dowfax 2a1 surfactant and temperature on the electroplating of Bi nanowires in ion-track etched polymer membranes**

While the addition of surfactant is absolutely necessary to homogeneously electrodeposit Bi in polycarbonate templates with interconnected nanochannels, it is not required for the production of nanowire arrays by electrodeposition in templates with parallel channels.^1^ Thus, by synthesizing Bi nanowires in templates with parallel channels both, with and without surfactant addition, we investigate whether and to which extent the surfactant significantly affects the growth process.^1, 2^  Figure S1 shows the mean current recorded during Bi pulse plating (*U*_ON_ = -200 mV, *t*_ON_ = 20 ms, *U*_OFF_ = -170 mV, *t*_OFF_ = 100 ms) in templates with nanochannel diameters between 60 and 170 nm. The potential *U* was measured versus a standard calomel electrode with saturated KCl solution inside. Every point in the graphs corresponds to the recorded current averaged over 10 consecutive pulses.^1^ The electrodeposition took place at both 22°C (a,b) and 40°C (c,d) using an electrolyte with (b,d) and without (a,c) the addition of a per mile of Dowfax 2a1 surfactant. A similar current-time (*I-t*) behavior is observed for platings at recorded at the same temperature with and without surfactant, indicating that the presence of surfactant does neither influence growth rate nor filling homogeneity when using templates with parallel channels.


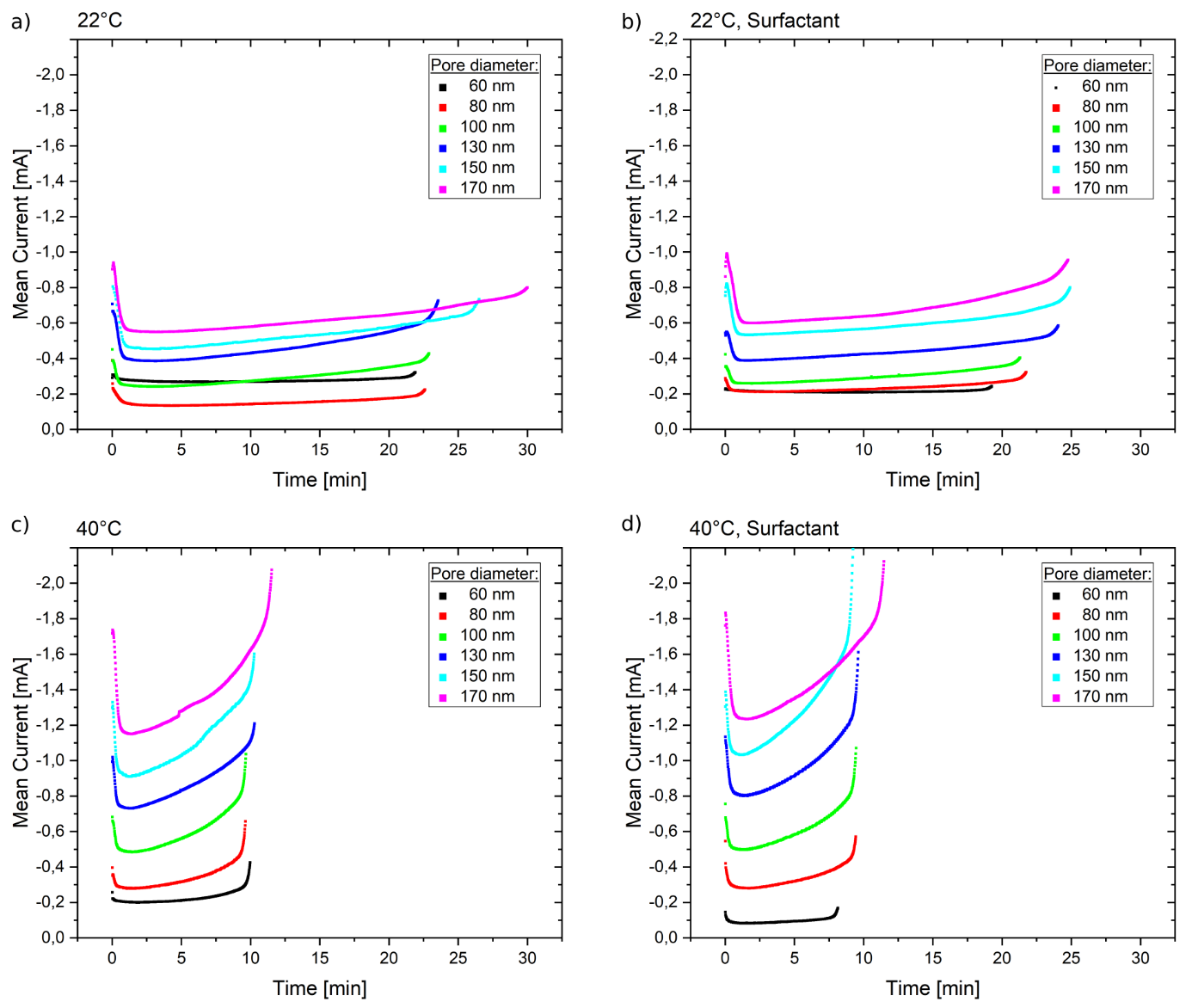


**Figure S1** Mean current vs. plating time for pulse platings a), c) without surfactant at 22°C and 40°C respectively and b), d) with surfactant at 22°C and 40°C, respectively. The sharp current increase at the end of each curve marks the moment when the material growing inside the template reaches the end of the pores and starts to form so called caps on top of the template.

This is contrary to previous electrodeposition experiments in templates with interconnected channels, strongly indicated that the presence of pore intersections in the networks templates and/or the various inclination of the channels with respect to the membrane surface hindered the homogeneous electrodeposition of Bi without addition of wetting agents.^1, 2^

### **Effects of surfactant Dowfax 2a1 and temperature on the crystallographic properties of Bi nanowires in ion-track etched polymer membranes**

Figure S2 displays the x-ray diffractograms of Bi nanowire arrays with wire diameter 60 nm (a,c) and 170 nm (b,d) electroplated using the same pulse plating conditions as for Fig. S1, but varying the surfactant concentration in the electrolyte between 0.00 and 2.00 ‰. The diffractograms did not reveal any systematic changes due to the surfactant concentration. The 60 nm diameter wire arrays exhibit a preferred {11.0} and {10.4} orientation in all cases, whereas the 170 nm diameter wires exhibit a strong {11.0} orientation with additional reflexions for several other planes, with the {10.4} planes being explicitly not observed.


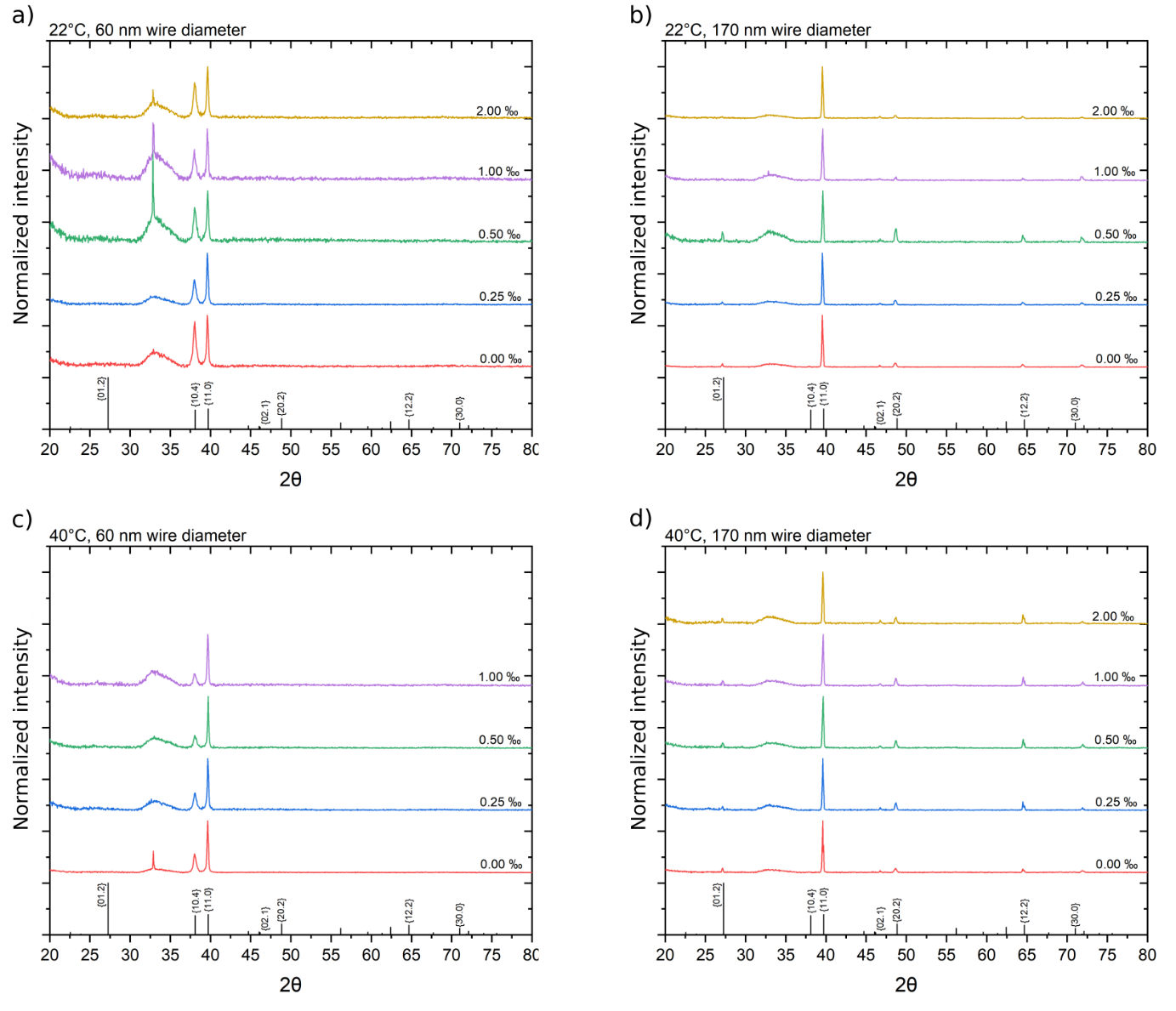


**Figure S2** Diffractograms of grown Bi nanowire arrays, electrodeposited with U_ON_ = -200 mV, t_ON_ = 20 ms, U_OFF_ = -170 mV, and t_OFF_ = 100 ms, with various surfactant contents in the electrolyte, recorded at 22°C (a,b) and 40°C (c,d), for wires with 60 (a,c) and 170 nm (b,d) diameter. The broad band between 30-35°, as well as the sharp reflexion at 32.8° stem from the Si sample holder.

TEM characterization.
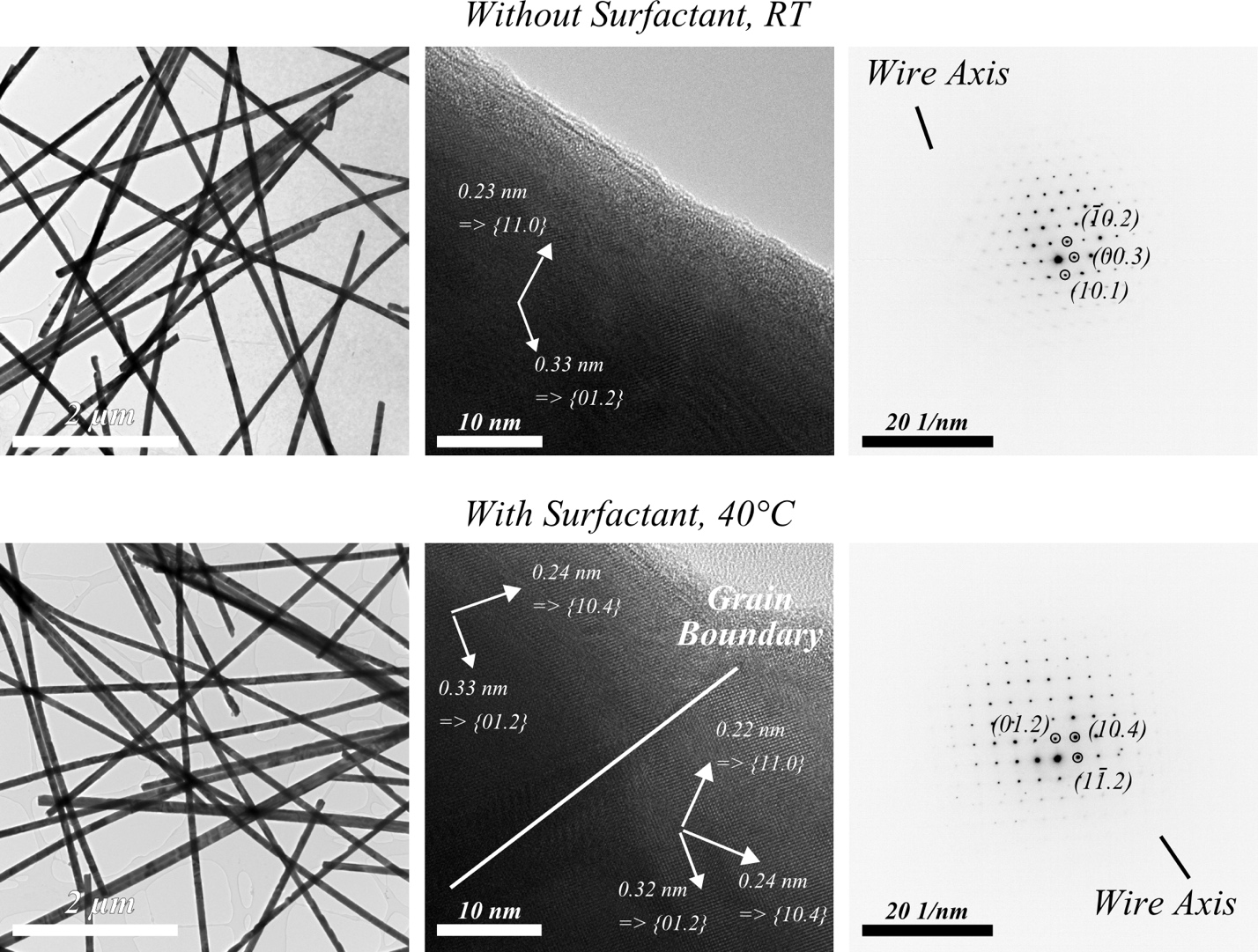


**Figure S3** Selected TEM and SAED images of 80 nm diameter Bi nanowires, grown without surfactant at room temperature and with a per mile surfactant at 40°C.

### **References**

1. Wagner, M. F. P., Völklein, F., Reith, H., Trautmann, C. & Toimil-Molares, M. E. Fabrication and thermoelectrical characterization of three-dimensional nanowire networks. *Phys. status solidi* **213**, 610–619 (2016).

2. Wagner, M. F. P. *et al.* Effects of Size Reduction on the Electrical Transport Properties of 3D Bi Nanowire Networks. *Adv. Electron. Mater.* **7**, 2001069 (2021).
